# Excess cost burden of diabetes in Southern India: a clinic-based, comparative cost-of-illness study

**DOI:** 10.1017/gheg.2016.2

**Published:** 2016-05-13

**Authors:** K. M. Sharma, H. Ranjani, A. Zabetian, M. Datta, M. Deepa, C. R. Anand Moses, K. M. V. Narayan, V. Mohan, M. K. Ali

**Affiliations:** 1 Vanderbilt University School of Medicine, Nashville, USA; 2 Madras Diabetes Research Foundation, Chennai, India; 3 Rollins School of Public Health, Emory University, Atlanta, USA; 4 Kilpauk Medical College and Hospital, Chennai, India

**Keywords:** Costs, diabetes, direct cost, economics, India, indirect cost

## Abstract

**Background:**

There are few data on excess direct and indirect costs of diabetes in India and limited data on rural costs of diabetes. We aimed to further explore these aspects of diabetes burdens using a clinic-based, comparative cost-of-illness study.

**Methods:**

Persons with diabetes (*n* = 606) were recruited from government, private, and rural clinics and compared to persons without diabetes matched for age, sex, and socioeconomic status (*n* = 356). We used interviewer-administered questionnaires to estimate direct costs (outpatient, inpatient, medication, laboratory, and procedures) and indirect costs [absence from (absenteeism) or low productivity at (presenteeism) work]. Excess costs were calculated as the difference between costs reported by persons with and without diabetes and compared across settings. Regression analyses were used to separately identify factors associated with total direct and indirect costs.

**Results:**

Annual excess direct costs were highest amongst private clinic attendees (INR 19 552, US$425) and lowest amongst government clinic attendees (INR 1204, US$26.17). Private clinic attendees had the lowest excess absenteeism (2.36 work days/year) and highest presenteeism (0.06 work days/year) due to diabetes. Government clinic attendees reported the highest absenteeism (7.48 work days/year) and lowest presenteeism (−0.31 work days/year). Ten additional years of diabetes duration was associated with 11% higher direct costs (*p* < 0.001). Older age (*p* = 0.02) and longer duration of diabetes (*p* < 0.001) were associated with higher total lost work days.

**Conclusions:**

Excess health expenditures and lost productivity amongst individuals with diabetes are substantial and different across care settings. Innovative solutions are needed to cope with diabetes and its associated cost burdens in India.

## Introduction

Diabetes is, in many ways, the quintessential chronic disease. It is a progressive disease leading to disabling and fatal complications, which are associated with increased costs. As of 2013, an estimated 382 million people worldwide are affected by diabetes, 80% of whom live in low- and middle-income countries where only 20% of global health expenditure on diabetes occurs [1]. In India alone, over 65 million adults have diabetes (one-sixth of the global diabetes population) and another 24.3 million people have prediabetes [1]. Further, only 19% of the Indian population is covered by central- or state-government sponsored insurance, leaving the majority of financing for diabetes to individuals in the form of out-of-pocket expenditure [2]. In addition, as young Indians with diabetes age and experience the medical complications of diabetes, the economic productivity of this segment of society may decline enough to significantly impact national economic productivity [2].

In the state of Tamil Nadu, diabetes affects 13.7% of urban and 7.8% of rural dwellers [3]. Increases in diabetes prevalence have been noted in both urban and rural regions over the prior three decades [3] and total costs associated with diabetes in India have risen in parallel with the rising prevalence. Total direct costs of urban diabetes doubled in India between 1998 and 2005 [4], largely due to complications as well increasing costs of care. Amongst patients hospitalized in Chennai between 2008 and 2009, those without diabetes related complications had significantly lower direct costs of hospitalization (INR 4493) than those with complications (INR 12690–INR 19020) [5]. At the household level, increased expenditures for diabetes have been manifest differently across the socioeconomic spectrum (SES), with lower socioeconomic strata households using a greater proportion of household income for diabetes care (urban poor 34% and rural poor 27%) [4].

Though there have been important advances in our understanding of the economic burdens of diabetes in India, significant gaps in our knowledge remain. Specifically, there are limited data regarding: (1) excess cost burdens of diabetes, as no reported study has compared costs of those with diabetes to those without diabetes [2, 4, 6–9]; (2) the indirect costs of diabetes in rural areas, where more than 70% of Indians live, where diabetes prevalence is increasing rapidly, and where healthcare spending and utilization patterns are known to be different [10–12]; and (3) a comprehensive valuation of indirect costs, with specific focus on the contributions of both presenteeism (decreased productivity for those who are unable to work as efficiently due to illness) and absenteeism (lost productivity caused by absence from work). Here we address these deficits and report on the direct and indirect excess costs of diabetes incurred across different SES groups in urban and rural settings in Southern India.

## Materials and methods

### Participants

In this individual perspective, clinic-based comparative cost-of-illness study in the Indian state of Tamil Nadu, self-reported costs amongst subjects with known diabetes were compared with subjects without known diabetes. From January to May 2010, individuals with diabetes (*n* = 606) were identified, recruited, and surveyed from three clinical sites: a government-funded tertiary-care urban clinic [Kilpauk Medical College (KMC) Hospital in Chennai] where the majority of clinic attendees are poor urbanites; a private tertiary-care urban clinic [Dr Mohan's Diabetes Specialities Centre (DMDSC) in Chennai] where the majority of patients are middle or upper class urbanites; and a private primary-care rural clinic [Sai Rural Diabetes Specialities Centre (SRDSC) in Chunampet] which is the only regional clinic near the village of Chunampet and also serves neighboring rural communities.

Patients were selected randomly based on their presence at the clinic on scheduled recruitment days. Only prevalent cases of diabetes were enrolled in our study group as attributing health-related expenditures to diabetes would not be possible for incident cases.

Age-, sex-, and SES-matched control subjects without diabetes (*n* = 356) were recruited from local communities that were proximal to recruitment sites and were likely to seek medical care at the respective recruitment site. We enrolled at least one control subject without diabetes for every two subjects with diabetes of the same age (±3 years), sex, and SES. For the urban population, SES matching was done based on presumptions of the relative affluence of the communities from which control subjects were recruited. For instance, control subjects for urban government clinic attendees were selected from an urban slum and control subjects for urban private clinic attendees were selected from middle and upper class neighborhoods. For the rural clinic, SES matching was based on random selections of control subjects from the village of Chunampet.

Inclusion criteria for subjects with diabetes were diagnosis of diabetes at least 12 months prior to the current visit, age greater than or equal to 18 years, and current residence in India. Inclusion criteria for subjects without diabetes were age greater than or equal to 18 years and no current or prior diagnosis of diabetes or prediabetes.

The study was approved by the Ethical Review Committee of the Madras Diabetes Research Foundation (MDRF). Informed consent was obtained from all study participants prior to solicitation of data.

No standardized instrument exists for assessing cost of illness, particularly for chronic diseases in developing countries. Therefore, we utilized an existing questionnaire that has been used in the Indian context [13] to solicit basic sociodemographic information and developed *de novo* questions to assess direct health expenditures and indirect costs related to diabetes. The questionnaire was reviewed by independent health economics experts and was pilot-tested among ten randomly selected clinic patients. Based on feedback regarding comprehension and ease of use, the questionnaire was amended accordingly. The English questionnaire is included in the appendix (online Supplementary Appendix A). The questionnaire was then translated to the vernacular (Tamil) by a certified translator and independently reverse-translated to English to verify accuracy. Four interviewers were trained over 1 week to ensure comprehension of each question and ability to effectively explain each question to a lay respondent in English or Tamil. All interviewers participated in multiple monitored trial administrations of the questionnaire. During the training program, the intra-observer agreement coefficient (kappa statistic) range was 0.70–1 and the inter-observer agreement coefficient range was 0.65–0.96.

### Data collection

Respondents were asked to recall health expenditures [in Indian Rupees (INR)] for the three months prior to their clinic presentation, not including the current visit. Measures of direct costs included outpatient and inpatient healthcare provider fees, medications, supplies, laboratory tests, transportation, special food(s), rehabilitation or physical therapy, and hired help for household activities.

Indirect costs included absenteeism (defined as the number of days absent from work in the prior three months) secondary to medical need (doctor's appointments, illness sustained at home, and hospitalizations) and presenteeism (decreased productivity while at work). Presenteeism was assessed by asking respondents to recall the number of days in the prior three months that they accomplished 25, 50, and 75% of their usual daily tasks in a standard work day. Three month recollection was chosen to maximize participant recall and to allow capture of recent incurred health-related expenditures.

### Analysis

Following data entry, data integrity was verified by random double entry of a subset of questionnaires with an acceptable error frequency of 0.5%.

Statistical analyses were conducted using SPSS for Windows version 19 (PASW Statistics for Windows, SPSS Inc, Chicago). All costs were reported in Indian Rupees (INR) and were also converted to US Dollars ($) with an appropriate conversion rate applied for the period of data collection (46 INR = 1$).

Annual total direct costs were calculated by summing all reported direct expenditures over 3 months and multiplying by 4. Total indirect costs were calculated by combining the total lost work days from absenteeism and presenteeism and multiplying this sum by the per diem economic value assigned to each individual [either self-reported income for earning individuals or self-reported replacement costs (fees that would be paid to outside help to replace the work of non-earning individuals)]. Excess costs were calculated by subtracting costs for subjects without diabetes from costs for subjects with diabetes. Since self-reported costs were not all normally distributed, these data were reported as means and standard deviations (in-text tables), and also as medians with inter-quartile ranges (Appendix Tables).

General linear regression models were built to investigate associations between participant characteristics [sociodemographic (e.g., age and income) and clinical factors (e.g., duration of diabetes)] with outcomes (total annual direct health expenditures and total working days lost). Income, direct cost, and lost work days were log-transformed due to their non-normal distribution. Interactions between the independent variables were also considered in our models. For continuous and categorical variables, estimated regression coefficients and their 95% confidence intervals (CIs) were reported.

## Results

Demographic and socioeconomic characteristics of 606 persons with diabetes and 356 individuals without diabetes, across settings, are described in Table 1. Mean age, sex distributions, household size, and annual income were similar for corresponding subjects with and without diabetes across settings. In urban government and rural clinics, participants had similar median annual incomes whereas respondents attending the urban private clinic had higher annual incomes. Comparing those with and without diabetes within each setting, the education level of the respondents and heads of households were significantly different. Within the urban private population, the household size (with DM: 4.23 persons *v.* without DM: 3.79 persons) and median incomes (with DM: INR 150 000 *v.* without DM: INR 240 000) were significantly different. Amongst rural dwellers, significant differences in general characteristics of people with diabetes compared with those without diabetes included higher mean age (with DM: 54.0 years *v.* without DM: 49.2 years); larger household size (with DM: 4.66 persons *v.* without DM: 3.74 persons); and greater income (with DM: INR 60 000 *v.* without DM: INR 42 000).

**Table 1.** Characteristics of participants with and without diabetes by setting

Setting	Government		Private		Rural	
	With DM	Without DM	With DM	Without DM	With DM	Without DM
Sample size	206	122	200	92	200	142
Age in years	53.7 (10.8)	53.3 (13.1)	55.4 (11.2)	52.3 (13.7)	**54.0 (11.1)**	**49.2 (15.8)**
% male	44	38	65	61	62	54
Household size	3.92 (1.8)	3.96 (1.8)	**4.23 (1.8)**	**3.79 (1.5)**	**4.66 (2.2)**	**3.74 (1.7)**
Education of respondent (%)						
1	**78.6**	**63.6**	**31**	**13**	**56.5**	**90.1**
2	**20.9**	**33.1**	**35**	**32.6**	**33**	**7.7**
3	**0.5**	**1.7**	**23.5**	**40.2**	**3**	**2.1**
4	**0.0**	**1.7**	**10.5**	**14.1**	**7.5**	**0.0**
Education of head of household (%)						
1	**70.9**	**49.2**	**25.5**	**7.6**	**42.5**	**85.2**
2	**27.7**	**47.5**	**37.5**	**29.3**	**42.5**	**12.7**
3	**1**	**2.5**	**23**	**43.5**	**5.5**	**1.4**
4	**0.5**	**0.8**	**14**	**19.6**	**9.5**	**0.7**
Median annual income in INR (IQR) [USD]	48 000 (48 000) [$1 043.5]	53 000 (24 000) [$1 152.2]	**150 000 (286 500) [$3 260.9]**	**240 000 (399 000) [$5 217.4]**	**60 000 (84 000) [$1 304.3]**	**42 000 (30 000) [$913.0]**

Data are presented as means (standard deviation) unless otherwise stated.

**Bold** text signifies *p* < 0.05; DM, diabetes mellitus; IQR, inter-quartile range.

Education scale: 1 = primary school or less completed, 2 = primary to higher secondary education completed, 3 = technical or graduate degree, 4 = post-graduate degree.

1 USD = 46.00 INR.

The absolute and excess direct costs for each setting are presented in Table 2 and Appendix B. Absolute annual direct costs amongst those with diabetes were greatest in the private clinic attendees [INR 20 684 ($449.70)], followed by rural [INR 16 484 ($358.40)] and government [INR 2184 ($47.50)] attendees. The annual excess costs (incremental expense differences between respondents with and without diabetes) were highest among those attending the private clinic [INR 19 552 ($425.04)] and lowest among those attending the government clinic [INR 1204 ($26.17)]. In the rural setting, excess direct costs of diabetes were more similar to those of private clinic attendees [INR 15 576 ($338.61) *v.* INR 19 552 ($425.04), respectively] despite reported income being closer to the population attending the government clinic. Excess costs due to diabetes were greater for outpatient than inpatient care, across all settings.

**Table 2.** Mean absolute and excess direct costs by setting

	Government		Private		Rural	
	With diabetes	Without diabetes	With diabetes	Without diabetes	With diabetes	Without diabetes
Absolute annual direct costs						
OP direct costs in INR in USD	1876 (8216.8)	980 (4852.9)	11 176 (22 565.5)	1132 (3988.6)	13 436 (13 268.4)	904 (2527.6)
	$40.80	$21.30	$243	$24.60	$292.10	$19.70
IP direct costs in INR in USD	308 (2591.7)	0 (0)	9508 (37 178.1)	0 (0)	3048 (25 877.1)	2 (16.7)
	$6.70	$0	$206.70	$0	$66.30	$0
Total direct costs in INR in USD	2184 (8892.7)	980 (4852.9)	20 684 (49 690.4)	1132 (3988.6)	16 484 (29 004.3)	906 (2527.2)
	$47.50	$21.30	$449.70	$24.60	$358.40	$19.70
Excess annual direct costs in INR (USD)						
OP direct costs	896 ($19.48)		10 044 ($218.35)		12 528 ($272.35)	
IP direct costs	308 ($6.70)		9508 ($206.70)		3048 ($66.26)	
Total direct costs	1204 ($26.17)		19 552 ($425.04)		15 576 ($338.61)	

Data are presented as means (standard deviation) unless otherwise.

OP, outpatient; IP, inpatient.

1 USD = 46 INR.

Absolute annual total work days lost were greatest in government clinic attendees (48.8), followed by private (44.1) and rural (43.6) clinic attendees (Table 3 and Appendix C). Private clinic attendees had the lowest excess absenteeism (2.36 work days/year) and highest excess presenteeism (0.06 work days/year) due to diabetes compared with other settings. Government and rural clinic attendees had negative excess presenteeism (−0.31 and −0.10 work days/year, respectively), meaning that presenteeism was greater in respondents without diabetes than in those with diabetes. Participants attending the government clinic reported the highest excess absenteeism (7.48 work days/year) and lowest presenteeism (−0.31 work days/year). The rural population had indirect costs of intermediate range compared with the two other groups.

**Table 3.** Mean absolute and excess indirect costs by setting

	Government		Private		Rural	
	With diabetes	Without diabetes	With diabetes	Without diabetes	With diabetes	Without diabetes
Absolute annual indirect costs						
Absenteeism loss, work days/year	42.4 (60.9)	34.8 (36.8)	39.2 (44.1)	36.8 (53.3)	35.2 (20.5)	29.2 (17.1)
Presenteeism loss, work days/year	6.4 (34.9)	6.8 (14.5)	4.9 (26.5)	4.8 (13.1)	8.4 (20.3)	8.4 (24.1)
Total work days/year lost	48.8 (68.8)	41.0 (66.33)	44.1 (51.3)	41.9 (79.7)	43.6 (36.7)	37.5 (31.5)
Excess annual indirect costs						
Absenteeism loss, work days/year	7.48		2.36		6.19	
Presenteeism loss, work days/year	−0.31		0.06		−0.10	
Total work days/year lost	7.7		2.42		6.09	
Total annual productivity lost in INR (USD)	1576 ($34.26)		2462 ($53.52)		1913 ($41.59)	

Data are presented as means (standard deviation) unless otherwise 1 USD = 46 INR.

1 USD = 46 INR.

Table 4 shows the results of linear regression analyses exploring the associations of age, gender, education level, income, and duration of diabetes with total direct costs and total lost work days for all respondents, regardless of site. Ten additional years of diabetes duration was associated with 11% higher direct costs (*p* < 0.001). Older age (*p* = 0.02) and longer duration of diabetes (*p* < 0.001) were associated with total lost work days, but the magnitude of associations were small. Income was not associated with direct costs or lost work days. Adding the interactions between independent variables to our original model did not change the results.

**Table 4.** Associations between participant characteristics and direct costs and work days lost

	Total direct costs^a^			Total lost work days^a^		
	*β* coefficient	95% confidence interval	*p*	*β* coefficient	95% confidence interval	*p*
Age	−0.01	(−0.03 to 0.01)	0.1	0.003	(0.000 to 0.006)	0.02
Male gender	0.49	(−1.57 to 2.56)	0.6	−0.22	(−0.58 to 0.13)	0.2
Education level 1^b^	−0.54	(−2.40 to 1.33)	0.5	−0.17	(−0.49 to 0.15)	0.2
Education level 2^b^	−0.22	(−2.14 to 1.70)	0.8	−0.07	(−0.40 to 0.26)	0.6
Education level 3^b^	−0.51	(−2.71 to 1.69)	0.6	−0.20	(−0.58 to 0.18)	0.3
Income^a^	−0.01	(−0.24 to 0.21)	0.9	−0.01	(−0.05 to 0.03)	0.5
Duration of diabetes	0.11	(0.08 to 0.14)	<0.001	0.01	(0.005 to 0.016)	<0.001

Education scale: 1 = primary school or less completed, 2 = primary to higher secondary education completed, 3 = technical or graduate degree, 4 = post-graduate degree.

a Log transformed.

b Education level 4 is the reference.

## Discussion

Foremost, our data from Southern India point to substantially higher per-capita cost burdens among individuals seeking care for diabetes than those not affected by diabetes. Secondly, these excess direct and indirect costs of diabetes are different across the socioeconomic and urban–rural spectrum in India. Such data on excess costs of diabetes are reported for the first time from India.

Based on recent prevalence estimates, total cost burdens of diabetes in India would amount to $32 billion, just over 2% of nominal gross domestic product (GDP) [4, 6]. Our data showed that the excess direct costs of diabetes varied from $26 to $425 annually, while the excess annual indirect costs varied from $34 to $53. Given that these are incremental costs of diabetes, they are reasonably consistent with published data from India showing total direct costs of $525.5 per person per year and indirect costs of $102.8 per person per year [6]. These are substantial numbers, particularly if one considers that $1 has three times the value in India in terms of purchasing power [14]. Secondly, national cost burdens due to diabetes are likely to rise with increasing prevalence. In addition, as the costs of health care services increase, per capita costs strictly related to diabetes may soon exceed the purchasing power of the average Indian. Lastly, our data point to significantly increased direct and indirect costs compared with studies completed just over a decade ago [7–9], suggesting increases in health care utilization and/or inflation.

Longer diabetes duration (but not age, gender, education, or income) was associated with higher total direct costs. Prior studies have confirmed similar associations between duration of diabetes and increased direct costs in India [4]. Our data are consistent with these findings with respect to incremental spending and add to this finding by exposing higher excess work days lost as well. Given that age alone did not explain higher direct costs, we can say with relative certainty that duration of diabetes and not simply increasing age is driving higher spending in the population we studied. Finally, the association of increased age and increased total lost work days is expected as older individuals with diabetes, a disease process that impairs immune competence [15] and general functioning, are more likely to have increased absenteeism and presenteeism compared with those without diabetes.

As expected, excess direct expenditures for diabetes vary considerably between urban *v.* rural settings and by SES groups within urban populations. The urban poor access care in government clinics, where the majority of expenditures are borne from public funds and out-of-pocket payments are minimal. As a result, lower-income urban (government) clinic attendees were the only subgroup with higher indirect costs associated with diabetes than direct expenditures.

Rural dwellers in Tamil Nadu had direct expenditures near those of the private dwellers, an unexpected finding in our study given that most rural Indians are less wealthy than urban middle and upper class Indians. This may reflect that the clinical site, Sai Rural Diabetes Specialities Centre (SRDSC), which is 40 km from Puducherry (a large city) and serves not only the local rural population, but also more affluent peri-urban and urban communities from neighboring regions and cities. As it is, the data are only generalizable to clinic attendees and possibly underrepresent those who cannot physically or financially access the clinic. While these results may not reflect the spending patterns of rural communities in a strict sense, they suggest that a segment of the rural population has significant spending power and that a comprehensive rural diabetes care clinic may have a more economically viable payer profile than previously thought. Also, given the dearth of nearby facilities for diabetes care, our site may have been the only location to obtain diabetes-related care, necessitating significant out-of-pocket spending, sale of personal assets, or borrowing to fund needed healthcare. Further study should be undertaken to probe the nature of resources used by rural citizens to fund diabetes care and generalizability about rural populations from our study is limited.

The vast majority of indirect costs in our study stemmed from absenteeism. Absentee days were least amongst the private cohort and nearly equal amongst rural and urban government clinic attendees; however, excess indirect costs were greatest for the private clinic sub-group because earnings are an order of magnitude greater than the other sub-groups. Fewer absentee days in the private sub-group might be related to middle and upper class occupations, which offer less flexibility from work-related absence during periods of illness. This may be further substantiated by the private cohort being the only sub-group to manifest more presenteeism in those with diabetes compared to those without diabetes.

The strengths of our study are manifold. To our knowledge, this study is the first to elucidate the excess costs of diabetes in urban and rural India, the first to investigate indirect costs in a rural population, and the first to attempt to tabulate presenteeism in the Indian context. Furthermore, both our excess direct and indirect cost data are of the same order of magnitude as recently published data from the same region in India, even though we have accounted for baseline levels of spending by individuals without diabetes [6]. These are powerful data that we hope will be impetus for significant change as outlined below.

Our study has limitations. Our matching methodology does not factor into account disease severity, prevalence of co-morbidities, education levels, and income between our diabetes and control subjects. This does limit our ability to calculate excess costs in a strict sense; however, we feel that we have an acceptable level of uncertainty when tabulating excess costs using our matched controls. Our controls are community-based, whereas our participants were selected in respective facilities; although necessary since we were recruiting participants from specific diabetes wards or hospitals, facility-based controls would be a more ideal control group. More rigorous matching is a welcomed challenge for future studies.

Further limitations include that there is still no standardized methodology for collecting and calculating direct or indirect cost data, so only broad generalizations of similarities can be made across various studies from India. Furthermore, income and cost data were skewed – to address this, we reported both means and medians and their associated measures of dispersal for added clarity. Our attempt to tabulate presenteeism was met with questionable success, as is evident by the estimates’ wide variance. Excess presenteeism was greater in those without diabetes compared with those with diabetes in two out of three sub-groups. This may have been a product of the difficulty in accurately communicating the concept of presenteeism to study participants. The questionnaire may also be improved to better elicit these data. Further study is needed to draw conclusions about presenteeism. However, given the prevalence of significant absolute (not excess) presenteeism, this entity should not be forgotten.

The focus on individual perspective, self-reported costs means that the expenses borne by the government for clinics attended by lower-income urban residents were not captured in our study, though these should be taken into account in any tabulation of total societal expenditure on diabetes. Indeed, lower expenditures by lower-income persons could be explained by the fact that poorer individuals forego recommended diagnostics or treatment because of inability to pay. Our reported costs also did not consider costs for those with undiagnosed diabetes. Though the comparison non-diabetes groups were people without known diabetes and were accessing care, some individuals may have had undiagnosed diabetes and incurred higher health costs than expected. Although this may be a limitation, the direction of this bias is toward the null (i.e. our data show a more conservative difference between groups, if anything). Arguably, the omitted costs may be even more substantial than those discussed here.

From a societal perspective, for example, we would want to calculate the indirect costs associated with premature mortality as diabetes accounts for an estimated 14.2% of all adult deaths in South Asia, with more than half of these deaths occurring in persons under the age of 60 [1]. Measuring these requires significant government buy-in and meticulous data collection, as well as considerable expense. Future research efforts should attempt to tabulate these. Additionally, our study may be limited by patients’ 3-month recall ability as we did not verify reported expenditures. Such data to verify self-reports were not available uniformly across the three recruitment sites. However, recognizing that longer recall periods lead to even greater recall bias, we utilized 3-month recall to minimize the bias. Furthermore, 5 years have elapsed since these data were collected. Based on trends in chronic disease costs, it is likely that our data underestimate the current costs of diabetes in India.

India is particularly poorly prepared to deal with the diabetes epidemic. Public spending on health provisions as a percentage of GDP – 0.94% – is among the lowest in the world, largely explaining the use of private out-of-pocket health expenditures, which account for 78% of all healthcare spending in India [10]. Between 1993–94 and 2004–05, despite a 67% increase in real per person income and an 82% increase in per person tax collections in India, real per person public health spending increased only 48% [10]. Furthermore, India has no public insurance scheme and only 10% of its population has medical insurance of any kind [10]. Its public and private health system is fraught with inefficiency and offers poor value for money in terms of quality and quantity of healthcare delivered [10]. Quality health care, even at high costs, is available only in large urban areas and not in smaller towns and rural areas where the majority of Indians reside. Lastly, there are challenges of corruption that siphon away money and resources intended to help the public, thereby further diminishing the efficacy of each rupee spent on health care [16, 17].

Numerous studies on costs of diabetes in India, including this one, point to an unsustainable trend of increasing numbers of individuals with diabetes and rising costs per individual. If the current trajectory continues unabated, India will quickly reach a precipice with a rapidly expanding number of individuals with diabetes receiving less frequent and lower quality care, leading to high morbidity and mortality [18–20]. Our study is a call for not only improved prevention efforts, but also innovative systems and financing solutions to better cope with impending financial obstacles related to diabetes care in India.

## Supplementary material

For supplementary material accompanying this paper visit http://dx.doi.org/10.1017/gheg.2016.2. click here to view supplementary material
