# Supplementary material for: Excess cost burden of diabetes in Southern India: a clinic-based, comparative cost-of-illness study
Source: Glob Health Epidemiol Genom. 2016 May 13;1:e8. doi: 10.1017/gheg.2016.2 (PMC5870439; doi:10.1017/gheg.2016.2)
Supplement: Supplementary file 1 [file S2054420016000026sup001.zip › S2054420016000026sup002.docx]

Appendix B: Median absolute and excess direct costs by setting

|  | | | Government | | | | Private | | | | Rural | | | |
| --- | --- | --- | --- | --- | --- | --- | --- | --- | --- | --- | --- | --- | --- | --- |
|  |  |  | With diabetes | | Without diabetes | | With diabetes | | Without diabetes | | With diabetes | | Without diabetes | |
| Absolute annual direct costs | OP direct costs in INR  in USD | 0 (265) | | 0 (0) | | 1440 (13740) | | 0 (0) | | 9564 (10455) | | 0 (200) | |  |
|  |  | $0 | | $0 | | $31.3 | | $0 | | $207.9 | | $0 | |  |
|  | IP direct costs in INR  in USD | 0 (0) | | 0 (0) | | 0 (0) | | 0 (0) | | 0 (0) | | 0 (0) | |  |
|  |  | $0 | | $0 | | $0 | | $0 | | $0 | | $0 | |  |
|  | Total direct costs in INR  in USD | 0 (600) | | 0 (0) | | 2100 (17550) | | 0 (0) | | 9624 (11779) | | 0 (260) | |  |
|  |  | $0 | | $0 | | $45.7 | | $0 | | $209.2 | | $0 | |  |
| Excess annual direct costs in INR (USD) | Total direct costs | 0 | | | | 2100 ($45.7) | | | | 9624 ($209.2) | | | |  |
| Data are presented as medians (interquartile range) unless otherwise  1 USD = 46 INR | | | | | | | | | | | | | | |

Appendix C: Median absolute and excess indirect costs by setting

|  | | Government | | Private | | Rural | |
| --- | --- | --- | --- | --- | --- | --- | --- |
|  | | With diabetes | Without diabetes | With diabetes | Without diabetes | With diabetes | Without diabetes |
| Absolute annual indirect costs | Absenteeism loss (work days/year) | 28 (0) | 28 (0) | 28 (4) | 28 (0) | 28 (8) | 28 (4) |
|  | Presenteeism loss (work days/year) | 0 (6) | 0 (0) | 0 (2.3) | 0 (0) | 0 (4) | 0 (4) |
|  | Total work days per year lost | 28 (10) | 28 (0) | 28 (12.3) | 28 (0) | 29 (16) | 28 (12) |
| Excess annual indirect costs | Total work days per year lost | 0 | | 0 | | 0 | |
| Data are presented as medians (interquartile range) unless otherwise  OP = outpatient; IP = inpatient  1 USD = 46 INR | | | | | | | |
