# Supplementary material for: Excess cost burden of diabetes in Southern India: a clinic-based, comparative cost-of-illness study
Source: Glob Health Epidemiol Genom. 2016 May 13;1:e8. doi: 10.1017/gheg.2016.2 (PMC5870439; doi:10.1017/gheg.2016.2)
Supplement: Supplementary file 1 [file S2054420016000026sup001.zip › S2054420016000026sup001.doc]

Costs Associated with Diabetes in Southern India

A. General & Identifying Information
1. Date of Interview ( dd / mm / yy ):	_____ / _____ / _____
2. Survey administered by:	_____________________________
3. Time questionnaire started:	_____________________________

4. What is your full name? ­­­­­­	_____________________________
5. What is your address:

Street Address

City

6. Pin Code:

7. What is (are) your phone number(s) – with area code:

Home:

| 9 | 1 | - |  |  |  |  |  |  |  |  |  |  |  |  |  |
| --- | --- | --- | --- | --- | --- | --- | --- | --- | --- | --- | --- | --- | --- | --- | --- |

Office:

| 9 | 1 | - |  |  |  |  |  |  |  |  |  |  |  |  |  |
| --- | --- | --- | --- | --- | --- | --- | --- | --- | --- | --- | --- | --- | --- | --- | --- |

Mobile:

| 9 | 1 | - |  |  |  |  |  |  |  |  |  |  |  |  |  |
| --- | --- | --- | --- | --- | --- | --- | --- | --- | --- | --- | --- | --- | --- | --- | --- |

8. M #: ______________________________
B. Medical Information
1. Have you ever had a major illness in the past?

⬜ Yes 🡪 complete table below	⬜ No 🡪 go to question 2

| Name of Illness | Year of illness | Duration of illness in days | Cured  (yes or no) | Still taking treatment (yes or no) |
| --- | --- | --- | --- | --- |
|  |  |  |  |  |
|  |  |  |  |  |
|  |  |  |  |  |
|  |  |  |  |  |
|  |  |  |  |  |
|  |  |  |  |  |

2. Have you ever had a major operation in the past?

⬜ Yes 🡪 complete table below	⬜ No 🡪 go to question 3

| Name of Operation | Indication | Year of operation | Cured  (yes or no) | Still taking treatment (yes or no) |
| --- | --- | --- | --- | --- |
|  |  |  |  |  |
|  |  |  |  |  |
|  |  |  |  |  |
|  |  |  |  |  |
|  |  |  |  |  |
|  |  |  |  |  |

3. Has a doctor ever told you that you have diabetes?

⬜ Yes 🡪 continue question 3	⬜ No 🡪 skip to question 4

a. If **yes**, when were you diagnosed?

Month: _______	Year: _______

b. If **yes**, are you currently taking treatment for diabetes?

⬜ Yes 🡪 complete parts b(i) and b(ii)	⬜ No 🡪 complete b(iii)

i. If **yes** to (b), how are you treating your diabetes?

⬜ Diet

⬜ Exercise

⬜ Insulin

⬜ OHA (medication)

⬜ Other;	specify: _______________

ii. If **yes** to (b), do you use any alternative medical treatment?

⬜ Yes

⬜ No

iii. If **no** to (b), why are you not taking treatment?

⬜ Not been instructed to

⬜ Too expensive / lack of funds

⬜ Have to travel too far to obtain treatment

⬜ Have to wait too long to obtain treatment

⬜ Too sick to manage medications

⬜ You forget

⬜ Side effects

⬜ Do not trust providers

⬜ Do not trust medication efficacy

⬜ Other; specify:	_______________

4. Has a doctor ever told you that you have high blood pressure?

⬜ Yes	⬜ No	⬜ Don’t know

a. If **yes**, for how long have you had high blood pressure?

_______ Months _______ Years

5. Has a doctor ever told you that you have diabetic kidney disease, sometimes referred to as protein in the urine or nephropathy?

⬜ Yes	⬜ No	⬜ Don’t know

a. If **yes**, for how long have you had kidney disease?

_______ Months _______ Years

6. Do you require dialysis?	⬜ Yes	⬜ No
   a. If **yes**, how many times per month do you currently receive dialysis?

_______ times per month

7. Have you ever had a kidney transplant?

⬜ Yes	⬜ No

a. Have you ever had any pain, discomfort, pressure, or heaviness in your chest?	⬜ Yes	⬜ No 🡪 Skip to (h)
b. Do you get pain in the center of the chest, in the left side of the chest, or the left arm?	⬜Yes	⬜ No 🡪 Skip to (h)
c. Do you get it when you walk at an ordinary pace on level or when you walk uphill or hurry?	⬜ Yes	⬜ No
d. Do you slow down if you get the pain while walking?

⬜ Yes	⬜ No

e. Does the pain go away if you stand still of if you take a tablet under the tongue?	⬜ Yes	⬜ No
f. Does the pain go away in less than 10 minutes?

⬜ Yes	⬜ No

g. Have you ever had a severe chest pain across the front of your chest lasting for half an hour or more?

⬜ Yes	⬜ No

NOTE: If answers to (c) – (g) are yes, then the patient may have angina or may have had a heart attack and needs referral. Contact your study supervisor and alert them of this response.

h. Have you ever had any of the following: difficulty in talking, weakness of arm and/or leg on one side of the body or numbness on one side of the body?

⬜ Yes	⬜ No

NOTE: If answer to (h) is yes, then the patient may have had a TIA or stroke and needs referral. Contact your study supervisor and alert them of this response.

9. Have you ever been diagnosed by a physician with numbness, tingling, or burning sensations in your feet or legs, also known as neuropathy?

⬜ Yes	⬜ No	⬜ Don’t know

a. If **yes**, for how long have you had this condition?

_______ Months _______ Years

10. Has a doctor ever told you that you have foot (pressure) ulcers or wounds on your feet that do not heal?

⬜ Yes	⬜ No	⬜ Don’t know

a. If **yes**, for how long have you had foot (pressure) ulcers?

_______ Months _______ Years

11. Have you ever had an amputation?

⬜ Yes	⬜ No

12. Has a doctor ever told you that you have eye disease or blurring of vision (retinopathy or cataracts)?

⬜ Yes	⬜ No	⬜ Don’t know

a. If **yes**, for how long have you had eye disease?

_______ Months _______ Years

b. Have you ever had cataract surgery or removal of a cloudy eye lens and replacement with a new lens?

⬜ Yes	⬜ No

c. If **yes**, have you ever had laser treatment (pan-retinal photocoagulation) for eye disease?

⬜ Yes	⬜ No

d. Are you currently blind in only one eye?

⬜ Yes	⬜ No

e. Are you currently blind in both eyes?

⬜Yes	⬜ No

i. If **yes** to (e), how long you have been blind?

_______ Months _______ Years

13. Has a doctor ever told you that you have had a stroke (weakness / paralysis on one side or a blood clot in the brain)?

⬜ Yes	⬜ No	⬜ Don’t know

a. If **yes**, how long has it been since you had your first stroke?

_______ Months _______ Years

b. If **yes**, how many strokes have you had in total?	_______ strokes

14. Have you ever required medical attention for a very low blood sugar (hypoglycemic) episode?

⬜ Yes	⬜ No	⬜ Don’t know

a. If **yes**, how many have you had in the previous 3 months?

_______ episodes

b. If **yes**, how many times have you sought medical attention for these in your lifetime?	_______ times

15. Have you ever required medical attention for a very high blood sugar (hyperglycemic) episode?

⬜ Yes	⬜ No	⬜ Don’t know

a. If **yes**, how many have you had in the previous 3 months?

_______ episodes

b. If **yes**, how many times have you sought medical attention for these in your lifetime?	_______ times

16. Please indicate if you currently use or previously used any of the products listed below.

| Form of tobacco | Reported Consumption | | Duration of use | | |
| --- | --- | --- | --- | --- | --- |
|  | Average amount (E.g.: 3 cigarettes) | Frequency –  1 = per day  2 = per week  3 = per month |  |  |  |
|  |  |  | Months | Years | Currently using? If no, year stopped. |
| Cigarettes (1 filtered) |  |  |  |  |  |
| Cigarettes(1 unfiltered) |  |  |  |  |  |
| Beedis (1) |  |  |  |  |  |
| Cigars (1) |  |  |  |  |  |
| Hookah |  |  |  |  |  |
| Pipe |  |  |  |  |  |
| Chewing tobacco |  |  |  |  |  |
| Snuff (by nose) |  |  |  |  |  |
| Ghutka or paan masala (1 packet) |  |  |  |  |  |
| Paan (1) |  |  |  |  |  |
| Betel nut (1 packet) |  |  |  |  |  |

17. Please indicate if you currently use or previously used any of the alcoholic beverages listed below.

| Form of alcohol | Reported Consumption | | Duration of use | | |
| --- | --- | --- | --- | --- | --- |
|  | Average amount (E.g.: 3 glasses wine) | Frequency –  1 = per day  2 = per week  3 = per month |  |  |  |
|  |  |  | Months | Years | Currently using? If no, year stopped. |
| Spirits (30 mL of brandy, gin, rum, vodka, whisky, etc.) |  |  |  |  |  |
| Wine (120 mL glass) |  |  |  |  |  |
| Beer (285 mL bottle) |  |  |  |  |  |
| Country liquor, arrack (30 mL measure) |  |  |  |  |  |
| Toddy (200 mL) |  |  |  |  |  |

18. Do you take any medicines regularly?

⬜ Yes	⬜ No 🡪 skip to question 19

a. If **yes**, please specify the following for each allopathic medication you take.

| Name of medication (generic or brand) | Drug class | Dosage (mg) | Prescribed | | Taken | | Unit MRP (Rs.) | # of doses per unit |
| --- | --- | --- | --- | --- | --- | --- | --- | --- |
|  |  |  | # | Freq* | # | Freq* |  |  |
|  |  |  |  |  |  |  |  |  |
|  |  |  |  |  |  |  |  |  |
|  |  |  |  |  |  |  |  |  |
|  |  |  |  |  |  |  |  |  |
|  |  |  |  |  |  |  |  |  |
|  |  |  |  |  |  |  |  |  |
|  |  |  |  |  |  |  |  |  |
|  |  |  |  |  |  |  |  |  |
|  |  |  |  |  |  |  |  |  |
|  |  |  |  |  |  |  |  |  |
|  |  |  |  |  |  |  |  |  |
|  |  |  |  |  |  |  |  |  |
|  |  |  |  |  |  |  |  |  |
|  |  |  |  |  |  |  |  |  |
|  |  |  |  |  |  |  |  |  |
|  |  |  |  |  |  |  |  |  |
|  |  |  |  |  |  |  |  |  |
|  |  |  |  |  |  |  |  |  |
|  |  |  |  |  |  |  |  |  |
|  |  |  |  |  |  |  |  |  |

*Frequency key: 1 = per day; 2 = per week; 3 = per month; 4 = per 3 months

b. If **yes**, please specify the following for each ayurvedic or homeopathic medication you take.

| Name of medication | Cost incurred **per month** (Rs.) |
| --- | --- |
|  |  |
|  |  |
|  |  |
|  |  |
|  |  |

19. If you are not taking medications as advised by a medical professional, please indicate why (select all that apply).

⬜ Not applicable

⬜ Cannot afford to	⬜ Side effects	⬜ Hard to access

⬜ Forget	⬜ Don’t need to	⬜ Don’t believe medicines work

20. From where do you most often obtain your medications (select all that apply)?

⬜ Private pharmacy	⬜ Private hospital pharmacy

⬜ Public hospital pharmacy	⬜ Clinics

⬜ Charitable sources	⬜ Other; specify: ___________________

21. Do you currently have health insurance?

⬜ Yes 🡪 please continue this question	⬜ No 🡪 skip to question 22

a. Please indicate the type of health insurance you have:

____________________

b. Please indicate which of the following is covered by your health insurance:

⬜ inpatient visits (hospitalizations)

⬜ operations / surgical care

⬜ outpatient visits

⬜ medicines (excluding insulin)

⬜ insulin

⬜ laboratory tests

⬜ transportation

⬜ diabetes supplies (glucometer, testing strips, syringes, etc)

⬜ physical therapy

⬜ paid assistance

22. Over the previous three months, from which of the following sources do you pay for your medical care and, if applicable, your diabetic care? Please indicate the relative percentages next to each option. Indicate ‘no expenditure’ if applicable.

|  |  | Percentage of total health care expenditure (%) | Percentage of diabetes care expenditure (%) |
| --- | --- | --- | --- |
| a. | Out-of-pocket |  |  |
| b. | Money borrowed from family or friends |  |  |
| c. | Money loaned commercially |  |  |
| d. | Health Insurance (including ESI, company sponsored insurance) |  |  |
| e. | Government provided |  |  |
| f. | Charitable sources |  |  |
| g. Other; specify |  |  |  |
| h. |  | ⬜ No expenditure | ⬜ No expenditure |

C. Direct Costs Information
1. Please respond to the following questions regarding **outpatient** care you received in the previous **three months**. Outpatient care is all instances of medical care that did not necessitate hospitalization.
   a. How many times did you seek outpatient care?	_______ times

|  | Item | Average cost incurred  (Rupees) | 1 – per day  2 – per week  3 – per month  4 – per 3 months  Frequency |
| --- | --- | --- | --- |
| b. | Doctor / nurse fees |  |  |
| c. | Medicines (including insulin) |  |  |
| d. | Supplies (including glucose strips, gauze, sterile solution, etc.) |  |  |
| e. | Laboratory tests |  |  |
| f. | Transportation (including ambulance fees) |  |  |
| g. | Special foods |  |  |
| h. | Physical exercise or therapy |  |  |
| i. | Hired help or a paid assistant |  |  |
| j. | Other out-of-pocket expenses  Specify: _______________ |  |  |
| k. | Don’t know; total aggregated costs |  |  |

2. Please respond to the following questions regarding **inpatient** care you received in the previous **three months**. Inpatient care is all instances of medical care that required you to be admitted to a hospital.
   a. How many times were you hospitalized?	_______ times

|  | Item | Average cost incurred  (Rupees) | 1 – per day  2 – per week  3 – per month  4 – per 3 months  5 – per visit  Frequency |
| --- | --- | --- | --- |
| b. | Doctor / nurse fees |  |  |
| c. | Medicines (including insulin) |  |  |
| d. | Supplies (including glucose strips, gauze, sterile solution, etc.) |  |  |
| e. | Laboratory tests |  |  |
| f. | Transportation (including ambulance fees) |  |  |
| g. | Special foods |  |  |
| h. | Physical exercise or therapy |  |  |
| i. | Paid assistance |  |  |
| j. | Other out-of-pocket expenses  Specify: |  |  |
| k. | Don’t know; total aggregated costs |  |  |

3. If applicable, list the number of visits and costs incurred (including medicines, laboratory tests, supplies, travel, etc.) in the outpatient and inpatient settings with respect to each of the following complications in the previous **three months**. The complication should have been the primary reason for you to seek medical attention.

|  | Condition | Number of outpatient visits | Total cost of outpatient visits (Rs.) | Number of inpatient visits | Total cost of inpatient visits (Rs.) |
| --- | --- | --- | --- | --- | --- |
| a. | High blood pressure |  |  |  |  |
| b. | High cholesterol |  |  |  |  |
| c. | Kidney disease (including dialysis) |  |  |  |  |
| d. | Heart disease |  |  |  |  |
| e. | Foot ulcers / sensation problems / neuropathy |  |  |  |  |
| f. | Amputations |  |  |  |  |
| g. | Stroke / paralysis |  |  |  |  |
| h. | Eye disease (retinopathy and cataracts) |  |  |  |  |
| i. | Hypoglycemic episode(s) |  |  |  |  |
| j. | Hyperglycemic episode(s) |  |  |  |  |

D. Indirect Costs Information
1. Are you currently employed?

⬜ Yes 🡪 skip to question 2	⬜ No 🡪 continue question 1

a. If **no**, are you unable to work or perform your typical duties for a medical reason (medically disabled)?

⬜ Yes 🡪 answer (i) – (iii)	⬜ No 🡪 skip to 2

i. If **yes** to (a), for how long have you been disabled?

_______ Months _______ Years

ii. If **yes** to (a) and you were earning prior to your disability, what was your income prior to your disability?

Rs. _______________ per day / week / month / year

iii. If **yes** to (a) but you were not earning an income prior to your disability, how much would hiring help to replace the work that you did routinely prior to your disability cost?

Rs. _______________ per day / week / month / year

2. In an average week of 7 days, how many days do you currently spend working on your primary occupation?

_______ Days

3. In the previous three months, how many days were you unable to perform your typical daily work activities (such as going to your office if you are a professional, working on a farm if you are a farmer, or working around the house if you are a housewife) for a **full day** because of illness or sickness in which you stayed home?

_______ Days

4. In the previous three months, how many days are you unable to perform your typical daily work activities for a **full day** because of illness or sickness for which you were hospitalized?

_______ Days

5. In the previous three months, how many days are you unable to perform your typical daily work activities for a **full day** because you were tending to your medical needs (going to see the doctor, obtaining supplies, etc.)?

_______ Days

6. Over the previous three months, please indicate how many days you have felt only partially as productive as you are normally. For instance, if you were only able to perform approximately 1 out of 4 of your typical daily tasks for 3 days in the previous 3 months, write ‘3’ next to completed 25% of your daily tasks. Then indicate the same information for 50% and 75% of your daily tasks.
   a. Completed 25% of your daily tasks:	_______ days
   b. Completed 50% your daily tasks:	_______ days
   c. Completed 75% of your daily tasks:	_______ days
7. Which of the following accounts for the primary reason that you were not able to be as productive as you normally are, as asked about in question 6? Please select the single biggest contributor.

⬜ Inability to concentrate	⬜ Fatigue	⬜ Illness	⬜ Lethargy

⬜ Pain	⬜ Other	⬜ Not applicable

8. In the previous three months, did another individual whom you did **not** pay for help assist you while you were ill or obtaining medical care?

⬜ Yes; continue this question	⬜ No; skip to section E

If **yes**, please indicate for how many days in the previous three months you have been accompanied in the following settings by the named persons, requiring that person to forego his/her typical daily activities for an entire day.

Please fill in the ‘other’ column if an individual helped you but is not described by a relationship below. Then please indicate the occupation for each person that helped you using the codes provided in the table. For all individuals with occupation listed as ‘other’, please indicate approximate annual earnings. For all non-earning individuals, please indicate how much one would have to pay to hire help to perform the work that the individual does in a typical day.

Also indicate if over the previous three months any of the individuals below had to work additional hours (including obtaining a new job) to supplement income due to your illness. If the individual worked a job different from the one normally worked, please indicate so as well.

|  | Father | Mother | Brother | Sister | Spouse | Child | Friend | Other 1; specify | Other 2; specify |
| --- | --- | --- | --- | --- | --- | --- | --- | --- | --- |
| Home (days) |  |  |  |  |  |  |  |  |  |
| Clinic or outpatient visit (days) |  |  |  |  |  |  |  |  |  |
| Hospitalization (days) |  |  |  |  |  |  |  |  |  |
| Operation (days) |  |  |  |  |  |  |  |  |  |
| Occupation (code) |  |  |  |  |  |  |  |  |  |
| Replacement costs for non-earners and ‘other’ per year (Rs.) |  |  |  |  |  |  |  |  |  |
| Total additional hours worked |  |  |  |  |  |  |  |  |  |
| New occupation, if applicable (code) |  |  |  |  |  |  |  |  |  |

Other 1’s relationship to you: ____________________

Other 2’s relationship to you: ____________________

Use the table below to code the appropriate number corresponding to occupation in the table above.

| Still in school / college / pursuing higher degree | 1 |
| --- | --- |
| Professional (doctor, engineer, lawyer, IT) | 2 |
| Teacher / professor | 3 |
| Clerical / office staff | 4 |
| Corporate employee: e.g. assistant manager, etc. | 5 |
| Corporate executive: e.g. manager and above | 6 |
| Homemaker | 7 |
| Laborer | 8 |
| Retired | 9 |
| Government | 10 |
| Farmer | 11 |
| Other | 12 |
| Unemployed | 13 |

E. Quality of Life

Please respond to one choice corresponding to each of the following categories.

1. Mobility

I have no problems in walking.	⬜

I have some problems in walking about.	⬜

I am confined to bed.	⬜

2. Self-Care

I have no problems with self-care.	⬜

I have some problems washing or dressing myself.	⬜

I am unable to wash or dress myself.	⬜

3. Usual Activities

I have no problems with performing my usual activities.	⬜

I have some problems with performing my usual activities.	⬜

I am unable to perform my usual activities.	⬜

4. Pain / Discomfort

I have no pain or discomfort.	⬜

I have moderate pain or discomfort.	⬜

I have extreme pain or discomfort.	⬜

5. Anxiety / Depression

I am not anxious or depressed.	⬜

I am moderately anxious or depressed.	⬜

I am extremely anxious or depressed.	⬜

6. To help people say how good or bad a health state is, we have drawn a scale (rather like a thermometer) on which the best state you can imagine is marked 100 and the worst state you can imagine is marked 0. We would like you to indicate on this scale how good or bad your health is today in your opinion. Please do this by drawing a line from the box below to whichever point on the scale indicates how good or bad your health state is today.

90

80

70

60

50

40

30

20

10

100

**Worst**

**imaginable**

**health state**

0

**Best**

**imaginable**

**health state**

Your health today

F. Willingness to Pay Information
1. Please respond to this question only if you are a diabetic.

If there were a treatment that could completely cure you of diabetes, what is the maximum amount you would be willing to pay for it?

Rs. __________

2. If there were a way to prevent diabetes, what is the maximum amount you would be willing to pay for it assuming you were not diabetic at the time of the payment?

Rs. __________

G. Demographic and Socioeconomic Information
1. What is your date of birth? ( dd / mm / yy ) _____ / _____ / _____
2. What is your age?	_______ years	_______ months
3. What is your gender?	⬜ Male	⬜ Female	⬜ Transgender
4. What is your religious affiliation (if any)?

⬜ Hindu / Hinduism

⬜ Muslim / Islam

⬜ Christian / Christianity

⬜ Sikh / Sikhism

⬜ Buddhist / Buddhism

⬜ Jain / Jainism

⬜ Jewish / Judaism

⬜ Parsi / Zoroastrianism

⬜ No religion / Atheist / Atheism

⬜ Spiritual

⬜ Other; specify: ___________________

⬜ Unknown / Not willing to state

5. What is your marital status?

⬜ Never Married	⬜ Widowed	⬜ Divorced	⬜ Separated	⬜ Married

6. What is the highest level of education you have completed? Please check only one option.

⬜ No formal schooling

⬜ Less than primary school (passed 1^st^ – 4^th^ standard)

⬜ Primary school completed (passed 5^th^ – 9^th^ standard)

⬜ High school completed (passed 10^th^ and 11^th^ standard)

⬜ Higher Secondary school completed (passed 12^th^ standard)

⬜ Technical education (e.g. ITI)

⬜ Undergraduate degree

⬜ PG degree or above

⬜ Prefer not to answer

7. What is the highest level of education completed by the head of your household? The head of the household is defined as the individual who is the primary earner or the individual who makes the majority of financial decisions. Please check only one option.

⬜ No formal schooling

⬜ Less than primary school (passed 1^st^ – 4^th^ standard)

⬜ Primary school completed (passed 5^th^ – 9^th^ standard)

⬜ High school completed (passed 10^th^ and 11^th^ standard)

⬜ Higher Secondary school completed (passed 12^th^ standard)

⬜ Technical education (e.g. ITI)

⬜ Undergraduate degree

⬜ PG degree or above

⬜ Prefer not to answer

8. What is the language medium in which you were educated in primary school?

⬜ Tamil	⬜ English	⬜ Hindi	⬜ Other; specify: _______________

9. How many members live in your household? A household is defined as all of those who share one kitchen.

_______ members

a. How many children are in your household?	_______
b. How many children are financially dependent on you?	_______
11. What is your household income?

Rs. _______________ per day / week / month / year

12. Do you earn an individual income?

⬜ Yes; answer part (a) & (b)	⬜ No; answer part (c)

a. Rs. _______________ per day / week / month / year
b. **Including yourself**, how many people do you support with your individual income?

_______

c. If ‘no’ to question 12, please indicate how much one would have to pay to hire help to replace the work that you do (E.g., If you are a housewife, how much one would have to pay to hire help to cook, clean, take care of children, etc.)?

Rs. _______________ per day / week / month / year

13. Regarding the location of your current and previous residence(s):

| a. | How do you classify where you currently live? | Urban area = 1  Rural area = 2 | |  |
| --- | --- | --- | --- | --- |
| b. | How would you classify the area where you spent the majority of your childhood? | Urban area = 1  Rural area = 2 |  |  |
| c. | How would you classify the area where you have spent the majority of your life? | Urban area = 1  Rural area = 2 | |  |

14. Please answer the following with respect to the facilities within your home:

| Facility | Types | | Score |
| --- | --- | --- | --- |
| a) House | Pucca  Semi pucca  Kutcha | 4  2  0 |  |
| b) Toilet facility | Own flush toilet  Shared flush toilet/own pit toilet  Shared/public pit toilet  No facility | 4  2  1  0 |  |
| c) Source of lighting | Electricity  Kerosene/ gas/ oil  Others | 2  1  0 |  |
| d) Main fuel for cooking | Electricity/LPG/biogas  Coal/charcoal/kerosene  Others | 2  1  0 |  |
| e) Drinking water source | Pipe, hand pump / well in residence/ Packaged water  Public tap/hand pump/well  Others | 2  1  0 |  |
| f) Separate room for cooking | Yes  No | 1 0 |  |
| g) House ownership | Yes  No | 2  0 |  |
| h) Agricultural land ownership | ≥ 5 acres  2-4.9 acres  <2 acres  No agricultural land | 4  3  2  0 | If answer is 0,  skip i and go to j |
| i) Irrigated land ownership | Yes  No | 2  0 |  |
| j) Livestock ownership  (Livestock that gives income to the family) | Cattle  Poultry  None | 4  2  0 |  |
| k) Ownership of durable goods  (Yes – 1, No – 2) | Car; Tractor; Moped/scooter/motorcycle; Mobile telephone; Refrigerator; Bicycle; Electric fan; Washing machine; Sewing machine; Television; Water pump; Bullock cart; Thresher Computer |  |  |

15. Time questionnaire completed:	_______________
